# Clinical Global Impression of Cariprazine in Negative Symptom Schizophrenia Patients: Comparison of clinical trial data vs. real-world evidence

**DOI:** 10.1192/j.eurpsy.2023.1333

**Published:** 2023-07-19

**Authors:** E. Rancans, Z. B. Dombi, R. Csehi, G. Németh

**Affiliations:** 1 Department of Psychiatry and Narcology, Riga Stradins University, Riga, Latvia; 2 Gedeon Richter Plc., Budapest, Hungary

## Abstract

**Introduction:**

There is an increasing need to understand the effectiveness of novel medications in real-world context since despite being the gold standard, double-blind trials have their own limitations as well. Clinical Global Impression is a simple tool for clinicians to assess the severity of an illness (CGI-Severity) as well as to rate how much the patient’s disorder has improved or worsened relative to baseline (CGI-Improvement). In this poster, cariprazine, a third-generation antipsychotic medication that was found to be effective in the treatment of negative symptoms in schizophrenia will be evaluated.

**Objectives:**

To compare the effectiveness of cariprazine in clinical trial vs real-world setting via the CGI-S and CGI-I scales in negative symptom schizophrenia patients.

**Methods:**

We compared the results of a clinical trial (Németh et al. Lancet 2017; 389:1103-13) and an observational study (Rancans et al. Int Clin Psychopharmacol. 2021;36(3):154-161). The latter was an open-label, flexible-dose, 16-week, observational study of cariprazine involving 116 outpatients in Latvia. Adult patients who have been diagnosed with schizophrenia, exhibited negative symptoms based on clinical judgement, were at least mildly ill according to the CGI-S scale and have not previously received cariprazine were eligible to take part in the study. Dosing of cariprazine was based on clinical judgement. The clinical trial was a randomized, double-blind, multi-centred, 26-week study with adults aged 18–65 years with long-term (>2 year), stable schizophrenia and predominant negative symptoms (>6 months). Patients were randomly assigned to monotherapy with cariprazine 4.5 mg/day or risperidone 4.0 mg/day.

**Results:**

116 patients on flexible dose cariprazine (observational study) were compared with 227 patients on cariprazine 4.5 mg/day and 229 on risperidone 4.0 mg/day (clinical trial). Baseline severity of illness as measured by the CGI-S was between moderately and markedly ill in all three groups. By the end of the 26-week trial, cariprazine reduced the CGI-S score significantly (LS Mean Change: -0.9, p<0.01). In contrast, the risperidone group achieved only -0.7-point change from baseline. In the observational study, cariprazine also achieved significant change (-0.9, p<0.001) but by week 16. In terms of improvement, patients on cariprazine improved minimally to much in both the clinical trial and real-world setting.

**Conclusions:**

The effectiveness of cariprazine in clinical trial and real-world setting do not seem to differ as measured by the scales in negative symptom patients.

**Disclosure of Interest:**

E. Rancans Grant / Research support from: Gedeon Richter, Lundbeck, Consultant of: Abbvie, Gedeon Richter, Grindex, Janssen Cilag, Lundbeck, Servier, Zentiva, Speakers bureau of: Abbvie, Gedeon Richter, Grindex, Janssen Cilag, Lundbeck, Servier, Zentiva, Z. Dombi Employee of: Gedeon Richter Plc., R. Csehi Employee of: Gedeon Richter Plc., G. Németh Employee of: Gedeon Richter Plc.

